# Acute Kidney Injury Is Associated with Higher Serum Cys-C and NGAL Concentrations, and Risk of Mortality in Premature Calves with Respiratory Distress Syndrome

**DOI:** 10.3390/ani13020232

**Published:** 2023-01-08

**Authors:** Merve Ider, Mahmut Ok, Amir Naseri, Alper Erturk, Tugba Melike Parlak, Ramazan Yildiz, Murat Kaan Durgut

**Affiliations:** 1 Department of Internal Medicine, Faculty of Veterinary Medicine, Selcuk University, Konya 42250, Turkey; 2 Department of Internal Medicine, Faculty of Veterinary Medicine, Hatay Mustafa Kemal University, Hatay 31060, Turkey; 3 Department of Pharmacology and Toxicology, Faculty of Veterinary Medicine, Selcuk University, Konya 42250, Turkey; 4 Department of Internal Medicine, Faculty of Veterinary Medicine, Burdur Mehmet Akif Ersoy University, Burdur 15030, Turkey

**Keywords:** acute kidney injury, premature calf, hypoxia, biomarkers

## Abstract

**Simple Summary:**

This study aimed to evaluate hypoxic acute kidney injury in premature calves with respiratory distress syndrome using kidney-specific biomarkers. Ten-term healthy calves and 70 premature calves with respiratory distress syndrome were included in the study. At admission and 72 h, arterial blood gas analysis to evaluate hypoxia and serum blood urea nitrogen, creatinine, phosphorus, cystatin-C, neutrophil gelatinase-associated lipocalin, uromodulin, and liver-type fatty acid-binding protein concentrations were measured for evaluation of kidney functions. Acute renal failure developed in 38.5% of premature calves with respiratory distress syndrome. The mortality risk in premature calves with acute renal failure was four times higher than in those without acute kidney injury. In addition, serum cystatin-C and neutrophil gelatinase-associated lipocalin concentrations were significantly higher in calves with acute kidney injury than those without. In conclusion, it causes acute renal failure in premature calves with respiratory distress syndrome. Cystatin-C and neutrophil gelatinase-associated lipocalin were found to be useful markers of hypoxic-acute kidney injury in premature calves with respiratory distress syndrome.

**Abstract:**

The purpose of the present study was to establish the development of acute kidney injury (AKI) and evaluate the usefulness of kidney-specific biomarkers in diagnosing AKI in premature calves with respiratory distress syndrome (RDS). Ten-term healthy and 70 premature calves with RDS were enrolled. Clinical examination, blood gases, and chemical analysis were performed at admission and 72 h. Serum concentrations of blood urea nitrogen (BUN), creatinine (Cre), phosphorus (P), cystatin-C (Cys-C), neutrophil gelatinase-associated lipocalin (NGAL), uromodulin (UMOD), and liver-type fatty acid-binding protein (L-FABP) were measured to evaluate kidney injury. Our findings showed that 38.5% of the premature calves with RDS developed AKI. The RDS-AKI group had a 4-fold higher mortality risk than the RDS-non-AKI group. Cys-C, with 90% and 89% specificity, and NGAL, with 100% sensitivity and 85% specificity, were the most reliable biomarkers to determine AKI in premature calves. The usefulness of any biomarker to predict mortality was not found to be convincing. In conclusion, AKI can develop as a consequence of hypoxia in premature calves and may increase the risk of mortality. In addition, serum Cys-C and NGAL concentrations may be useful in the diagnosis of AKI in premature calves with RDS.

## 1. Introduction

Respiratory distress syndrome (RDS) and its related complications are the most important causes of premature calf deaths [1,2,3,4,5]. Respiratory distress syndrome in premature calves is characterized by increased respiratory effort, insufficient oxygen exchange, and hypoxia [3,4,5]. Inadequate lung maturation and development of RDS in premature infants have been reported to be one of the most important causes of acute kidney injury (AKI) [6,7]. It has been reported that the incidence of AKI in newborn infants with RDS is 25–66% [8]. Co-occurrence of RDS and AKI in newborn infants contributes significantly to the increase in mortality rate [9,10,11].

Respiratory distress syndrome in newborns is considered one of the main causes of pre-renal failure because it reduces glomerular filtration rate (GFR) and renal blood flow. Since the kidneys are susceptible to a decrease in blood oxygen concentration, hypoxia due to RDS can lead to AKI within 24 h [12,13]. Premature infants have fewer nephrons and a low GFR, which may make them more susceptible to AKI and nephrotoxicity [13,14].

Evaluation of kidney functions in premature infants is essential to ensure the safe dosing of drugs and to detect AKI at an early stage [15]. Biochemical analyses based on creatinine (Cre) and blood urea nitrogen (BUN) were insufficient for accurate and early diagnosis of AKI. Therefore, interest in biomarker studies such as cystatin C (Cys-C), neutrophil gelatinase-associated lipocalin (NGAL), uromodulin (UMOD), and liver-type fatty acid binding protein (L-FABP) has increased in recent years for the determination of AKI [6,16].

Cystatin-C is a cysteine protease inhibitor synthesized by nucleated cells [7,16]. Previous studies in premature infants with RDS showed that serum Cys-C enables the detection of AKI development at an earlier stage than Cre [7], and high serum Cys-C concentration in preterm newborns with RDS is associated with the development of AKI [17]. Neutrophil gelatinase-associated lipocalin is expressed in many organs, such as the kidney, stomach, lung, and colon. It has been defined that plasma and urinary NGAL concentrations increase in ischemic or nephrotoxic kidney injury, and it is also the most reliable biomarker for the earliest detection of hypoxic AKI [16,18,19]. Uromodulin, also known as Tamm–Horsfall glycoprotein, is a glycoprotein that occurs physiologically in the urine and is released into the serum in small amounts [20]. It is stated that UMOD has a protective role in the development of AKI, and its concentrations decrease in AKI [21,22]. It has been reported that urinary L-FABP concentrations in premature infants with AKI were significantly higher than in term infants [23].

Although many studies [3,5,24,25,26,27] evaluated the function of organs such as lungs, intestines, and heart in premature calves, no study was found to evaluate renal function in premature calves. The present study was designed with the hypothesis that Cys-C, NGAL, UMOD, and L-FABP biomarkers, which are used in the diagnosis of AKI in premature infants, can be used to evaluate kidney function and diagnosis of AKI in premature calves with RDS. The purposes of the present study were to establish the development of AKI and the evaluation of the usefulness of kidney-specific biomarkers in the diagnosis of AKI in premature calves with RDS.

## 2. Materials and Methods

The study protocol was approved by the Institutional Ethics Committee of the Faculty of Veterinary Medicine, Selcuk University (No. 2020/93), and conducted from December 2020 to July 2022.

### 2.1. Healthy Calves

A convenience sample of 10 healthy calves (Holstein), >280 days gestation, and within the first 0–12 h after parturition were enrolled in the study. Calves were determined to be healthy based on clinical examinations and laboratory findings [28,29]. The calves were naturally born in faculty farm and could stand within 1 h and were fed 2 L of colostrum within the first 2 h of life. Calves from dystocia, prematurity, congenital abnormalities, and infection suspicion were excluded from the study.

### 2.2. Premature Calves with RDS

A convenience sample of 70 premature calves (39 Holstein, 21 Simmental, 5 Brown Swiss, 3 Belgian Blue, and 2 Charolais) with a gestation age between 230 and 260 days were admitted to the Large Animal Hospital of the Faculty of Veterinary Medicine, Selcuk University were enrolled in the study. All the calves included in the study were within the first 0–12 h after parturition. The criteria for prematurity were reduced gestation period, low body weight, a short silky hair coat, incomplete eruption of incisors, soft hooves, and weak or no sucking reflex. At admission, all premature calves had signs of respiratory distress syndrome (RDS). All premature calves were hospitalized for 3 days and received standard support, which included oxygen therapy and a feeding protocol following admission to the neonatal intensive care unit [3,27,29].

### 2.3. Clinical Examination

Clinical examinations of all calves included in the study were performed at admission and 72 h. In this context, hydration status, palpable lymph nodes, mucous membranes, capillary refilling time, mental status, and posture were evaluated. Heart and respiratory rate and rectal temperatures were measured.

### 2.4. Criteria for Definition of Respiratory Distress Syndrome (RDS)

The criteria for RDS were respiratory acidosis, hypoxia (PaO_2_ < 60 mmHg), hypercapnia (PaCO_2_ > 45 mmHg), tachypnea (respiratory rate > 45/min), abdominal respiration, and hyperlactatemia (>6 mmol/L) [3,27,29]. Among these parameters, the presence of at least two criteria along with PaO_2_ < 60 mmHg was taken into consideration [1,26,27].

### 2.5. Design of Study Groups

We conducted two prospective case-control studies. First, premature calves with RDS were divided into survivor group (n = 47) and non-survivor group (n = 23) according to their survival status. AKI biomarker concentrations were compared between healthy term calves (n = 10), survivors (n = 47), and non-survivor (n = 23) premature calves with RDS. In the second, the same biomarkers were compared between healthy term calves, premature calves with RDS who developed AKI (RDS-AKI subgroup), and those without AKI (RDS-non-AKI subgroup). The premature calves with RDS group were divided into two subgroups according to the development of AKI. The criteria for AKI in premature calves with RDS were creatinine (Cre), blood urea nitrogen (BUN), and phosphorus (P) levels. Calves with high levels of Cre (>2.2 mg/dL), BUN (>25 mg/dL), and phosphorus (>8 mg/dL) were included in the RDS-AKI subgroup (n = 27), and those within the normal reference range were included in the RDS-non-AKI subgroup (n = 43) [30].

### 2.6. Collection of Blood Samples

Blood samples were collected from the calves at the time of admission and 72 h. Blood samples for serum biochemistry analysis and biomarkers of renal injury were taken from the *Vena jugularis* and for blood gas measurement from *Arteria auricularis*. Non-anticoagulant tubes were used for serum collection. Sodium heparin-containing plastic syringes were used for blood gas measurement. Blood samples taken for biochemical analyses were kept at room temperature for 15 min, then centrifuged at 20× *g* for 10 min. Sera were removed and stored at −80 °C. Blood gas measurements were performed within 5 to 10 min of collection.

#### 2.6.1. Blood Gas Analysis

Arterial blood pH, partial oxygen pressure (PaO_2_), partial carbon dioxide pressure (PaCO_2_), oxygen saturation (SO_2_), lactate, glucose, sodium (Na), potassium (K), calcium (Ca), chlorine (Cl), bicarbonate (HCO_3_), and base deficit (BE) measurements were performed using an automatic blood gas analyzer (ABL 90 Flex, Radiometer, Brea, CA, USA).

#### 2.6.2. Biochemical Analysis

Blood urea nitrogen, Cre, and P levels of serum samples were measured using an automatic biochemistry machine (BT-3000 plus, Instrumentation Laboratory Company, Milan, Italy) [30].

#### 2.6.3. Evaluation of Brain-Related Biomarkers

Serum Cys-C, NGAL, UMOD, and L-FABP (ELK Biotechnology Co., Ltd., Wuhan, China) concentrations were measured with commercial bovine-specific ELISA test kits by the manufacturer’s instructions. Bovine Cys-C commercial ELISA kit (ELK Biotechnology Co., Ltd., Wuhan, China, Lot: 20334264377), bovine NGAL commercial ELISA kit (ELK Biotechnology Co., Ltd., Wuhan, China, Lot: 20334270272), bovine UMOD commercial ELISA kit (ELK Biotechnology Co., Ltd., Wuhan, China, Lot: 20334270272), bovine L-FABP commercial ELISA kit (ELK Biotechnology Co., Ltd., Wuhan, China, Lot: 20334270274) were used for ELISA analyzes of biomarkers. The intra-assay coefficient of variation (CV), inter-assay CV, and minimum detectable concentrations (MDC) for biomarkers were <8%, <10%, and 33 pg/mL for Cys-C, <8%, <10%, and 0.66 ng/mL for NGAL, <8%, <10%, and 0.263 ng/mL for UMOD, < 8%, <10%, and 0.58 ng/mL for L-FABP, respectively.

### 2.7. Statistical Analysis

SPSS 25 (IBM Corp^®^, 2017, Armonk, NY, USA) statistical program was used to evaluate the data. The Kolmogorov–Smirnov test was used to determine the variables’ normality and the variances’ homogeneity. Parametric data were expressed as mean ± SD and were evaluated using one-way analysis of variance (ANOVA) and the post hoc Tukey test, and Student’s t-test. Nonparametric data were expressed as median (minimum/maximum) and were evaluated using the Kruskal–Wallis test. Categorical data were analyzed with Chi-Square test. The Spearman correlation test was used to determine the correlation between variables. Receiver operating characteristic (ROC) analysis was performed to determine the prognostic cut-off value, sensitivity, and specificity of variables in non-survivor and survivor premature calves with RDS. In addition, the same test was used to evaluate whether AKI-related biomarkers have diagnostic significance. Statistical significance was considered as *p* < 0.05.

## 3. Results

### 3.1. Clinical Findings

Seventy (39 Holstein, 21 Simmental, 5 Brown Swiss, 3 Belgian Blue, and 2 Charolais) premature calves with RDS were included in the study. The mean body weight of the calves was 23.62 ± 4.83 in the RDS group and 45.71 ± 2.92 in the healthy term group. Of the premature calves with RDS, 47 (67%) survived, and 23 (33%) died. Seventeen of the premature calves died within the first 24 h, and six within 36 h. All (100%) healthy term calves in the control group survived. It was determined that 27 (38.5%) of the premature calves developed AKI. AKI developed in 13 (27.7%) of 47 surviving premature calves and 14 (60.9%) of 23 non-survivor calves. The mortality risk in RDS-AKI premature calves was four times (*p* = 0.007) higher than in premature calves with RDS-non-AKI (Table 1).

### 3.2. Blood Gas Analysis

At the time of admission, pH, PaO_2_, SO_2_, BE, and HCO_3_ levels of the survivor and non-survivor premature calves were significantly lower, and PaCO_2_ and lactate levels were higher than the healthy term calves (*p* < 0.05). Moreover, at the time of admission, the pH and SO_2_ levels of the non-survivor premature calves were lower, and the PaCO_2_ and lactate levels were higher than the survivor premature calves (*p* < 0.05). Also, pH, PaO_2_, SO_2_, Na, Glu, BE, and HCO_3_ levels increased significantly in the survivor premature calves at 72 h, and PaCO_2_ and lactate concentrations decreased (*p* < 0.05) (Table 2).

### 3.3. Biochemical Analysis

Serum biochemical analyses of premature and healthy term calves are presented in Table 3. Serum Cre levels of survivor and non-survivor premature calves were significantly higher than the healthy term calves (*p* < 0.05) and decreased at 72 h in the healthy term calves and survivor premature calves compared to the time of admission (*p* < 0.05). At the time of admission, BUN and P levels of non-survivor premature calves were higher than the control group (*p* < 0.05). In addition, the BUN levels increased in survivor premature calves at 72 h (*p* < 0.05).

### 3.4. Renal Biomarker Analysis for Evaluation of AKI

Renal biomarker analysis for the evaluation of AKI in healthy term calves and premature calves with RDS is presented in Table 4. In the RDS-AKI group, serum Cys-C and NGAL concentrations were significantly higher (*p* < 0.001) compared to the RDS-non-AKI group and healthy term calves. Serum L-FABP concentrations were higher in RDS-AKI and RDS-non-AKI groups than in healthy calves (*p* < 0.001).

**Figure 1:**
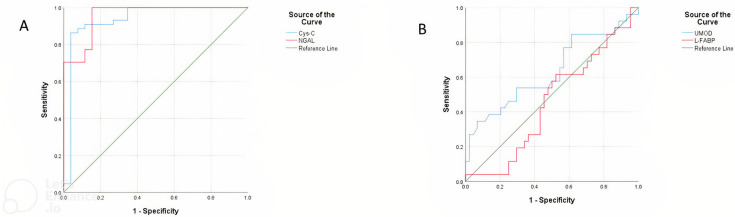
Receiver operating characteristic curve (ROC) analysis for the diagnosis of AKI premature calves with RDS based on serum concentrations of Cys-C, NGAL (**A**), UMOD, and L-FABP (**B**).

The results of the performed ROC analysis to distinguish the best biomarkers for detecting kidney injury in premature calves with RDS showed that Cys-C concentration at the cut-off point of 398.38 pg/mL, area under the curve (AUC) 0.934 (95% confidence interval (CI) 0.859–1.000; *p* < 0.001), with 90% sensitivity and 89% specificity, NGAL concentration at a cut-off point of 10.02 ng/mL, area under the curve (AUC) 0.957 (95% confidence interval (CI) 0.913–1.000; *p* < 0.001), with 100% sensitivity and 85% specificity were the most eligible markers to the diagnosis of AKI (Table 5; Figure 1A). The UMOD and L-FABP concentrations were not found to be convincing in the diagnosis of AKI (Figure 1B).

### 3.5. Renal Biomarker Analysis in the Healthy Term, Survivor, and Non-Survivor Premature Calves

Serum renal biomarker concentrations in the premature and healthy calves are presented in Table 6. Serum NGAL and L-FABP concentrations of survivor and non-survivor premature calves with RDS were significantly higher at the time of admission compared to the healthy term calves, while serum Cys-C concentrations were higher only in the non-survivor group (*p* < 0.05). Serum Cys-C, NGAL, and L-FAP concentrations of the survivor premature calves were higher than the control group at 72 h (*p* < 0.05). Serum NGAL concentrations of survivor premature calves decreased at 72 h compared to the time of admission, while serum UMOD and L-FABP concentrations were increased (*p* < 0.05). Also, in healthy term calves, serum UMOD concentration increased significantly at 72 h compared to the time of admission (*p* < 0.05).

**Figure 2:**
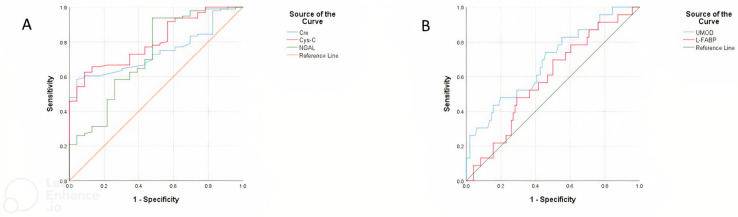
Receiver operating characteristic curve (ROC) analysis for the differentiation between the survivor and non-survivor premature calves with RDS based on the serum Cre, Cys-C, NGAL (**A**), UMOD, and L-FABP (**B**) concentrations.

The results of the performed ROC analysis to distinguish the best biomarkers for predicting mortality in premature calves with RDS are presented in Table 7. Our findings showed that Cys-C at the cut-off point of 375.27 pg/mL, area under the curve (AUC) 0.802 (95% confidence interval (CI) 0.717–0.887; *p* < 0.001) with 72% sensitivity and 66% specificity; NGAL at the cut-off point of 7.22 ng/mL, area under the curve (AUC) 0.721 (95% confidence interval (CI) 0.597–0.846; *p* = 0.001) with 62% sensitivity and 66% specificity (Figure 2A), UMOD at the cut-off point of 1.76 ng/mL, area under the curve (AUC) 0.685 (95% confidence interval (CI) 0.563–0.807; *p* = 0.006) with 60% sensitivity and 60% specificity, and Cre at the cut-off point of 3.41 mg/dL, area under the curve (AUC) 0.742 (95% confidence interval (Cl) 0.652–0.831; *p* < 0.001) with 66% sensitivity and 57% specificity were found to be significant prognostic indicators in premature calves with RDS (Figure 2B).

### 3.6. Correlation Analysis

**Figure 3:**
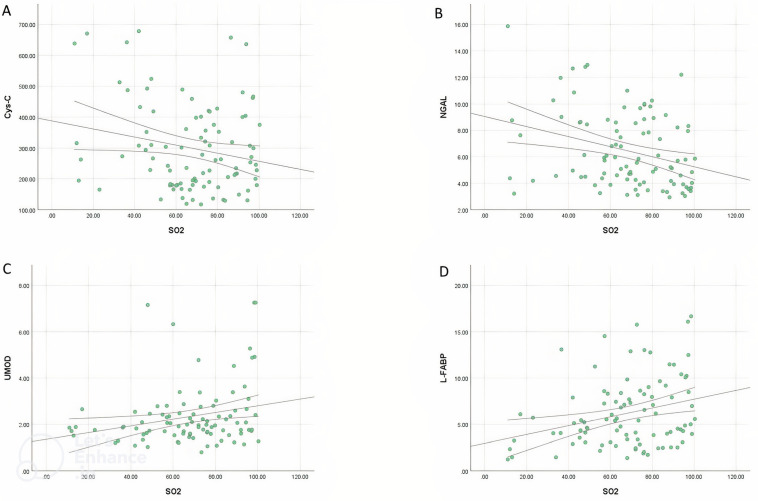
Correlation analysis graphs between arterial blood SO_2_ levels and Cys-C (**A**), NGAL (**B**), UMOD (**C**), and L-FABP (**D**) concentrations in premature calves with RDS.

The correlation between some arterial blood gas parameters and Cys-C, NGAL, UMOD, L-FABP, and Cre concentrations was presented in Table 8. Blood SO_2_ has a negative correlation with serum Cys-C concentration (*p* < 0.05; Figure 3A). Serum NGAL concentration was a negative correlation with PaO_2_ (*p* < 0.01) and SO_2_ (*p* < 0.01; Figure 3B) and positively correlated with PaCO_2_ (*p* < 0.05), lactate (*p* < 0.01), and Cre (*p* < 0.01). There was a negative correlation between serum UMOD concentration and blood PaCO_2_ (*p* < 0.01) and Cre (*p* < 0.01) levels and a positive correlation with PaO_2_ (*p* < 0.01) and SO_2_ (*p* < 0.05; Figure 3C). Serum L-FABP concentration was negatively correlated with blood SO_2_ levels (*p* < 0.01; Figure 3D).

## 4. Discussion

The present study evaluated the concentrations of renal injury biomarkers Cys-C, NGAL, UMOD, and L-FABP in the blood serum of newborn healthy term calves and premature calves with RDS. Our results showed that hypoxia-induced AKI develops in premature calves with RDS, and AKI was the most important predictor of mortality. Also, blood serum Cys-C and NGAL concentrations can be used to diagnose AKI.

It has been reported that the most important cause of mortality in premature calves is RDS, characterized by severe respiratory acidosis, hypercapnia, and hypoxemia [3,5,26,27]. Earlier studies conducted in newborn premature infants [11,12,31] stated that AKI is commonly developed in association with RDS and is one of the most important causes of mortality. It has been reported that 18% of premature infants develop AKI, and 42% of infants who develop AKI die [11]. A retrospective study conducted on 105 premature infants with RDS determined that 21 (20%) of the infants developed AKI, and the mortality rate was 61.9% [31]. In the present study, AKI developed in 27 (38.5%) of 70 premature calves with RDS. Of the 70 premature calves with RDS, 47 (67%) survived, while 23 (33%) calves died. AKI developed in 13 (28%) of 47 survivors and 14 (61%) of 23 non-survivor premature calves with RDS. The mortality risk in premature calves with AKI was four times (*p* = 0.007) more than in non-AKI premature calves. These findings indicate that the development of AKI with RDS is an important indicator of mortality in premature calves.

The most important changes in blood gases in premature calves with RDS are hypercapnia, hypoxia, hyperlactatemia, and mixed acidosis [1,3,4,26,27]. Consistent with previous studies [1,3,4,26,27], we found that at the time of admission, pH, PaO_2_, SO_2_, BE, and HCO_3_ levels of survivor and non-survivor premature calves with RDS were significantly lower, and PaCO_2_ and lactate levels were higher than the healthy term calves (*p* < 0.05). In addition, at the time of admission, the pH and SO_2_ levels of the non-surviving premature calves were lower, and PaCO_2_ and lactate levels were higher compared to the surviving premature calves (*p* < 0.05). These findings show significant changes in blood gases and acid-base balance due to hypoxia in premature calves with RDS. 

Today, the diagnosis of AKI in veterinary medicine is routinely made by an increase in serum Cre level and a decrease in urea excretion [32]. One of the important indicators of AKI is hyperphosphatemia, which develops due to the acute decrease in the number of functional nephrons and urinary phosphorus excretion due to insufficient compensation [33]. Blood urea nitrogen is less sensitive because it is affected by nonrenal factors such as high dietary protein intake and gastrointestinal bleeding. Therefore, serum Cre, which is considered more specific than BUN, is used more frequently in the diagnosis of AKI in newborns [17,32]. Our study used serum Cre, BUN, and P levels to establish AKI subgroups in premature calves with RDS. Serum Cre concentration of surviving and non-surviving premature calves was significantly higher than the healthy term calves (*p* < 0.05). Also, in healthy term calves and surviving premature calves, Cre levels were found to be decreased at 72 h compared to the time of admission (*p* < 0.05). Physiologically, creatinine crosses the placenta and reflects maternal renal function within 24–48 h of life. In addition, premature infants have higher serum Cre levels because of the immaturity of renal functions, low creatinine clearance, and increased reabsorption from immature tubules [14,15]. Consistent with the findings of previous studies, at the time of admission, higher Cre levels in the healthy term calves were associated with maternal Cre concentrations, and its high levels in premature calves were attributed to the immaturity of renal functions, low creatinine clearance and increased reabsorption from immature tubules.

In recent years, although Cys-C, NGAL, UMOD, and L-FABP biomarkers have been used to evaluate kidney functions in newborn premature infants in human medicine, no study has been found in veterinary medicine. Unlike Cre in infants, serum Cys-C concentrations are not affected by maternal Cys-C concentration, gender, gestational age, and muscle mass. Therefore, it is stated that Cys-C is superior to serum Cre for the evaluation of renal functions and GFR in the first days of life [7,9,16]. Serum Cys-C concentrations in premature infants with AKI-RDS were higher than in term healthy infants and non-AKI premature infants with RDS [9]. The authors showed that in the first 72 h of the life of premature infants, serum Cys-C had a 100% sensitivity and 83.3% specificity to predict AKI. They conclude that serum Cys-C is an important marker for the diagnosis of AKI in premature infants with RDS. Similarly, serum Cys-C concentrations at day 3 of life in premature infants with RDS-AKI were found to be higher than in RDS-non-AKI and control group and accentuated to be an independent predictor of AKI in premature infants with RDS [34]. In addition, it was determined that Cys-C concentrations increased in the umbilical cord blood of newborn infants with asphyxia, concerning the parameters indicating the severity of hypoxia/asphyxia [35]. In the present study, Cys-C concentrations were significantly higher in premature calves with RDS-AKI than RDS-non-AKI and healthy calves (*p* < 0.001), and a negative correlation was determined between blood SO_2_ levels. Also, Cys-C concentrations with 90% sensitivity and 89% specificity were determined to be an important indicator of AKI. Moreover, in a recent retrospective study, Cys-C was found to be higher in non-surviving acute RDS patients compared to survivors, and higher Cys-C concentrations at admission were strongly associated with 60-day mortality [36]. In the present study, serum Cys-C concentrations were significantly higher in the non-surviving premature calves with RDS compared to the survivors and healthy term calves with 72% sensitivity and 66% specificity. Consequently, serum Cys-C in premature calves with RDS may be a useful biomarker in the prediction of AKI, but it is not reliable in the prediction of mortality due to its low sensitivity and specificity.

In response to hypoxic-ischemic AKI, it has been found that renal tubular epithelial cells are damaged, and NGAL concentrations increase in serum and urine [19]. In addition, it is revealed that in preterm infants, NGAL is produced from immature nephrons and stimulates the growth of renal epithelium and glomerulogenesis [37]. In preterm infants, urinary NGAL concentrations are found to be high at birth due to ongoing renal maturation and decrease with advancing age [38,39]. In the present study, NGAL concentrations were significantly higher (*p* < 0.001) in the RDS-AKI group compared to RDS-non-AKI and healthy calves. Also, NGAL concentrations with 100% sensitivity and 85% specificity were found to be an important indicator of AKI prediction. Similar to our study, Surmiak et al. [40] found higher NGAL and lactate concentrations and lower pH and HCO_3_ levels in the asphyxiated AKI group than in non-AKI and healthy newborns. In our study, high serum NGAL concentrations and correlations between NGAL and blood gases parameters in the RDS-AKI group were associated with the development of AKI due to renal hypoxic-ischemic damage [19,40] and ongoing renal maturation [37,38,39]. According to these results, NGAL was evaluated as a reliable biomarker in the diagnosis of AKI in premature calves with RDS.

In a study conducted on premature infants, high urinary NGAL concentrations in preterm infants with AKI were independently associated with mortality [21]. Kayaaltı et al. [41] showed that plasma and urine NGAL concentrations were closely associated with mortality in patients with AKI. In the present study, at the time of admission, NGAL concentrations of the survivor and non-survivor premature calves were significantly higher than the healthy term calves (*p* < 0.05). Although NGAL concentration decreased at 72 h compared to the time of admission in survivor premature calves, it remained higher than the healthy calves (*p* < 0.05). Also, the NGAL concentrations with 62% sensitivity and 66% specificity were found to be a significant indicator of mortality. These findings demonstrated that NGAL concentrations can be used to estimate AKI in premature calves with RDS, but it is not a satisfactory marker for mortality prediction.

Uromodulin is a marker produced only from epithelial cells of the ascending limb of the loop of Henle (TAL) and epithelial cells of the early distal convoluted tubule. Because TAL epithelial cells are sensitive to oxidative stress, hypoxia, and nephrotoxins, UMOD concentrations are affected in medullary hypoxic kidney injury [42,43,44]. Although most of the studies on UMOD focus on the urinary form of UMOD, it has been shown that serum UMOD concentrations act in the opposite way (negative correlation) with renal filtration markers such as Cys-C, Cre, and BUN [45]. Studies have shown that serum UMOD concentrations decrease concerning acute and chronic kidney failure severity and have a protective role in kidney injury [21,22,46]. In the present study, although the serum UMOD concentrations were higher in the healthy calves than the premature calves with RDS, no statistically significant difference was found between the groups. In addition, serum UMOD concentrations correlated negatively with PaCO_2_ (*p* < 0.01) and Cre (*p* < 0.01) and positively with PaO_2_ (*p* < 0.01) and SO_2_ (*p* < 0.05). These findings indicated that serum UMOD concentrations in premature calves with RDS are associated with hypoxia and oxygenation of renal tissues, especially nephrons. In addition, serum UMOD concentrations of surviving premature and healthy term calves increased at 72 h compared to the time of admission (*p* < 0.05). In our opinion, this increase in newborn calves may reflect the increase in the number of functional nephrons and kidney mass associated with age, as described in experimental studies on TAL epithelial cells [42,43,44].

Previous studies on chronic renal failure have determined that low serum UMOD concentrations are associated with mortality, and high serum UMOD concentrations reduce the risk of mortality and renal failure [47,48]. However, in the present study, UMOD concentrations with 60% sensitivity and 60% specificity were found to be an important prognostic indicator (*p* = 0.006); the low sensitivity and specificity of this marker in predicting mortality significantly reduced its utility.

Liver-type fatty acid binding protein is expressed in the curved and straight parts of the proximal tubules of the kidneys in humans and animals [49]. It is excreted in the urine in the ischemic damage, low capillary blood flow, and oxidative stress that develops in the renal tubules [50]. It has been reported that L-FABP has a protective effect on the kidneys by accelerating fatty acid metabolism via β-oxidation and excretion of lipid peroxidation products from epithelial cells [51]. In addition, in mice with kidney dysfunction, FABP4 concentrations increase in blood circulation due to the inability to excrete enough into the urine [52]. However, it has been reported that serum L-FABP concentrations do not reflect renal damage, and urinary L-FABP concentration is more important in the diagnosis of renal failure [49,53]. Urinary L-FABP levels in preterm infants with AKI were significantly higher than in term infants [23]. It has been stated that proximal tubular damage and oxidative stress may effectively increase urinary L-FABP levels in preterm infants [54]. In the present study, serum L-FABP concentrations were higher in premature calves with RDS compared to the healthy term calves, and L-FABP concentrations increased at 72 h in survivor premature calves with RDS (*p* < 0.05). High L-FABP concentration in premature calves with RDS was evaluated as a protective response against hypoxia and oxidative stress in immature kidneys [54,55,56]. Ok et al. [4] determined that oxidative product increased in premature calves with RDS due to hypoxia, and antioxidant capacity decreased significantly. In light of previous studies, it is possible to conclude that the reason for the high serum L-FABP concentration may be related to the formation of oxidative stress [4], the excessive release of L-FABP into the blood to protect kidney function, and the inability to excrete it sufficiently in the urine due to the immature kidney structure [52].

Our study has some limitations. Firstly, the presence of histopathological findings provided the opportunity to prove the presence of acute renal failure at the microscopic level. Secondly, kidney-related biomarkers were evaluated in serum samples in the present study. If the measured biomarkers were determined in the urine of newborn calves as in human medicine, it would provide more precise information about the production and excretion of these markers. Additionally, as this is a case-control study, there is a high risk of spectrum bias.

## 5. Conclusions

It was determined that hypoxia and RDS might lead to AKI in premature calves, and the development of AKI significantly increases the mortality risk. In addition, serum Cys-C and NGAL concentrations in premature calves with RDS have satisfactory levels of sensitivity and specificity and may be a candidate for the diagnosis of AKI in premature calves with RDS.

## Figures and Tables

**Table 1:** The percentage of AKI and risk of mortality in premature calves with RDS.

Variable.	Non-AKI with RDS (n = 43)	AKI with RDS (n = 27)	*p*-Value	Odds Ratio (CI: 95%)
Survivors (n = 47)	72%	28%	0.007	4.068 (1.41–11.66)
Non-survivors (n = 23)	39%	61%		

**Table 2:** Arterial blood gas parameters of healthy term calves and premature calves with RDS.

Variable		Healthy Term Calves (n = 10)	Premature Calves with RDS		*p*-Value
			Survivors (n = 47)	Non-Survivors (n = 23)	
pH	Admission	7.40 ± 0.03 ^a^	7.18 ± 0.13 ^b^	7.02 ± 0.20 ^c^	
	72 h	7.47 ± 0.04	7.38 ± 0.09		<0.001
	*p*-Value	0.002	<0.001		
PaCO_2_ (mmHg)	Admission	36.65 ± 5.06 ^c^	56.42 ± 7.58 ^b^	69.46 ± 17.33 ^a^	
	72 h	36.78 ± 8.21	42.86 ± 9.21		0.056
	*p*-Value	0.966	<0.001		
PaO_2_ (mmHg)	Admission	72.19 ± 12.42 ^a^	29.26 ± 7.61 ^b^	26.62 ± 7.78 ^b^	
	72 h	69.34 ± 22.95	41.12 ± 15.56		0.004
	*p*-Value	0.734	<0.001		
SO_2_ (%)	Admission	95.24 ± 3.32 ^a^	56.44 ± 22.02 ^b^	33.57 ± 22.94 ^c^	
	72 h	92.76 ± 9.94	79.43 ± 16.27		0.016
	*p*-Value	0.464	0.000		
K (mmol/L)	Admission	4.08 ± 0.59	3.80 ± 0.66	3.63 ± 0.49	
	72 h	3.72 ± 0.56	3.84 ± 0.82		0.575
	*p*-Value	0.182	0.826		
Na (mmol/L)	Admission	148.10 ± 2.68	147.23 ± 7.34	148.08 ± 6.12	
	72 h	149.60 ± 9.27	153.02 ± 11.69		0.329
	*p*-Value	0.629	0.005		
Glu (mg/dL)	Admission	88.10 ± 28.13	58.97 ± 40.71	74.86 ± 56.30	
	72 h	100.10 ± 22.67	79.46 ± 24.01		0.022
	*p*-Value	0.308	0.004		
Lac (mmol/L)	Admission	4.37 ± 1.64 ^b^	7.02 ± 4.94 ^b^	11.32 ± 4.94 ^a^	
	72 h	1.73 ± 0.83	2.50 ± 1.74		0.053
	*p*-Value	0.001	<0.001		
BE (mmol/L)	Admission	−1.50 ± 2.82 ^a^	−5.84 ± 7.93 ^ab^	−10.22 ± 12.22 ^b^	
	72 h	3.10 ± 4.77	0.81 ± 4.91		0.186
	*p*-Value	0.017	<0.001		
HCO_3_ (mmol/L)	Admission	23.10 ± 2.83 ^a^	19.56 ± 5.16 ^ab^	17.19 ± 5.34 ^b^	
	72 h	26.86 ± 5.33	25.44 ± 4.44		0.449
	*p*-Value	0.065	<0.001		

PaCO_2_ (partial arterial carbon dioxide pressure), PaO_2_ (partial arterial oxygen pressure), SO_2_ (oxygen saturation), K (potassium), Na (sodium), Ca (calcium), Cl (chlorine), Glu (glucose), Lac (lactate), BE (base deficit), HCO_3_ (bicarbonate). Different letters (^a, b, c^) in the same line are statistically significant (*p* < 0.05).

**Table 3:** Serum biochemical analysis of healthy term calves and premature calves with RDS.

Variable		Healthy Term Calves (n = 10)	Premature Calves with RDS		*p*-Value
			Survivors (n = 47)	Non-Survivors (n = 23)	
BUN (mg/dL)	Admission	20.70 ± 6.50 ^b^	29.27 ± 13.29 ^ab^	31.63 ± 13.26 ^a^	
	72 h	18.58 ± 7.54	37.76 ± 21.78		<0.001
	*p*-Value	0.510	0.024		
Cre (mg/dL)	Admission	1.79 ± 0.37 ^b^	4.32 ± 2.19 ^a^	5.37 ± 4.44 ^a^	
	72 h	0.99 ± 0.09	2.08 ± 1.12		<0.001
	*p*-Value	<0.001	<0.001		
P (mg/dL)	Admission	7.12 ± 1.17 ^b^	7.04 ± 1.33 ^b^	9.92 ± 3.13 ^a^	
	72 h	7.91 ± 1.28	7.57 ± 1.81		0.495
	*p*-Value	0.168	0.110		

BUN (blood urea nitrogen), Cre (creatinine), P (phosphorus). Different letters (a, b) in the same line are statistically significant (*p* < 0.05).

**Table 4:** Biomarker concentrations result in healthy term, RDS-non-AKI, and RDS-AKI premature calves.

Variable	Healthy Term Calves (n = 10)	RDS-non-AKI (n = 43)	RDS-AKI (n = 27)	*p*-Value
Cys-C (pg/mL)	200.92 ^a^ (140.92–265.60)	264.72 ^a^ (119.17–492.31)	532.49 ^b^ (131.48–1448.39)	<0.001
NGAL (ng/mL)	3.86 ^a^ (3.29–5.99)	6.02 ^a^ (3.22–9.80)	11.97 ^b^ (7.47–18.44)	<0.001
UMOD (ng/mL)	1.82 (122–4.20)	1.76 (0.80–2.93)	1.58 (0.75–3.63)	0.178
L-FABP (ng/mL)	2.27 ^a^ (1.33–4.25)	5.06 ^b^ (1.46–13.63)	4.65 ^b^ (1.23–10.66)	0.001

Cys-C (cystatin C), NGAL (neutrophil gelatinase-associated lipocalin), UMOD (uromodulin), L-FABP (Liver-type fatty acid binding protein). Different letter on the same line indicated significant difference (*p* < 0.05) between the groups.

**Table 5:** The area under the curve (AUC), standard error, confidence interval (95%), cut-off values, respective sensitivity, and specificity of AKI prediction in premature calves with RDS.

Variable	AUC	Standard Error	*p*-Value	Asymptotic 95% Confidence Interval		Sensitivity	Specificity	Cut-Off Value
				Lower Band	Upper Bound			
Cys-C (pg/mL)	0.934	0.038	<0.001	0.859	1.000	90	89	398.38
NGAL (ng/mL)	0.957	0.023	<0.001	0.913	1.000	100	85	10.02
UMOD (ng/mL)	0.632	0.073	0.066	0.490	0.744	57	50	1.76
L-FABP (ng/mL)	0.455	0.070	0.527	0.317	0.592	53	53	4.83

Cys-C (cystatin C), NGAL (neutrophil gelatinase-associated lipocalin), UMOD (uromodulin), L-FABP (Liver-type fatty acid binding protein).

**Table 6:** Renal biomarker concentrations result in healthy term, survivor, and non-survivor premature calves.

Variable		Healthy Term Calves (n = 10)	Premature Calves with RDS		*p*-Value
			Survivors (n = 47)	Non-Survivors (n = 23)	
Cys-C (pg/mL)	Admission	204.54 ± 35.11 ^b^	324.74 ± 158.96 ^b^	531.61 ± 293.19 ^a^	
	72 h	198.33 ± 42.86	279.17 ± 121.27		0.001
	*p*-Value	0.727	0.117		
NGAL (ng/mL)	Admission	4.11 ± 0.88 ^b^	7.71 ± 2.98 ^a^	9.65 ± 4.27 ^a^	
	72 h	3.51 ± 0.34	5.34 ± 2.10		0.009
	*p*-Value	0.060	<0.001		
UMOD (ng/mL)	Admission	2.05 ± 0.92	1.83 ± 0.55	1.59 ± 0.62	
	72 h	3.07 ± 1.12	2.77 ± 1.64		0.482
	*p*-Value	0.039	<0.001		
L-FABP (ng/mL)	Admission	2.35 ± 0.77 ^b^	5.35 ± 2.70 ^a^	5.08 ± 2.94 ^a^	
	72 h	2.38 ± 1.36	6.97 ± 4.23		<0.001
	*p*-Value	0.945	0.029		

Cys-C (cystatin C), NGAL (neutrophil gelatinase-associated lipocalin), UMOD (uromodulin), L-FABP (Liver-type fatty acid binding protein). Different letters (^a, b^) in the same line are statistically significant (*p* < 0.05).

**Table 7:** The area under the curve (AUC), standard error, confidence interval (95%), cut-off values, respective sensitivity, and specificity of mortality prediction in non-survivor premature calves.

Variable	AUC	Standard Error	*p*-Value	Asymptotic 95% Confidence Interval		Sensitivity	Specificity	Cut-Off Value
				Lower Band	Upper Bound			
Cys-C (pg/mL)	0.802	0.044	<0.001	0.717	0.887	72	66	375.27
NGAL (ng/mL)	0.721	0.064	0.001	0.597	0.846	62	66	7.22
UMOD (ng/mL)	0.685	0.062	0.006	0.563	0.807	60	60	1.76
L-FABP (ng/mL)	0.587	0.063	0.196	0.464	0.710	60	54	4.95
Cre (mg/dL)	0.742	0.046	<0.001	0.652	0.831	66	57	3.41

Cys-C (cystatin C), NGAL (neutrophil gelatinase-associated lipocalin), UMOD (uromodulin), L-FABP (Liver-type fatty acid binding protein), Cre (creatinine).

**Table 8:** Spearman correlation analysis between serum renal biomarkers and some arterial blood gas parameters in premature calves with RDS.

Variable	PaCO_2_	PaO_2_	SO_2_	Lac	Cre
Cys-C (pg/mL)	0.017	−0.029	−0.207 *	0.189	0.191
NGAL (ng/mL)	0.253 *	−0.285 **	−0.301 **	0.327 **	0.396 **
UMOD (ng/mL)	−0.216 *	0.267 **	0.246 *	−0.201	−0.357 **
L-FABP (ng/mL)	−0.149	0.192	0.290 **	−0.092	−0.786

Cys-C (cystatin C), NGAL (neutrophil gelatinase-associated lipocalin), UMOD (uromodulin), L-FABP (Liver-type fatty acid binding protein), PaCO_2_ (partial arterial carbon dioxide pressure), PaO_2_ (partial arterial oxygen pressure), SO_2_ (oxygen saturation), Lac (lactate). * *p* < 0.05, ** *p* < 0.01.

## Data Availability

Not applicable.
